# Growth Hormone Concentrations in Different Body Fluids Before and After Moderate Exercise

**DOI:** 10.1186/s40798-016-0054-z

**Published:** 2016-08-11

**Authors:** Liz Gough, Lindy M. Castell, Rosalba Gatti, Richard J. Godfrey

**Affiliations:** 1 Centre for Human Performance, Exercise and Rehabilitation, Brunel University, Uxbridge, UK; 2 Nuffield Department of Anaesthetics, University of Oxford, Oxford, UK; 3 Green Templeton College, University of Oxford, Oxford, UK; 4 Endocrinology Unit, Azienda Ospedaliera, University of Padua, Padua, Italy

**Keywords:** Growth hormone, Saliva, Urine, Moderate exercise

## Abstract

**Background:**

Growth hormone (GH) has many direct and indirect actions and roles including substrate regulation and priming of some cells of the immune system, and the expected aspects of growth and repair. Different concentrations in human body fluids reflect the exercise-induced growth hormone response (EIGR) after exercise. In populations such as elite athletes, the invasive nature of venous sampling is poorly accepted. Thus, this study examines possible viable alternatives such as urine and saliva samples and the GH concentration.

**Methods:**

A heterogeneous group of 11 males (age 26.0 ± 5.0 years; body mass 76.5 ± 9.3 kg; VO_2peak_ 57.0 ± 6.0 mL kg^−1^ min^−1^) ran for 40 min on a treadmill at 5 % below their individually indentified lactate threshold pace. Samples of urine, saliva and blood were collected immediately pre- and post-test and at 30 and 60 min post-test.

**Results:**

Salivary GH was correlated with serum pre- and post-exercise (*p* < 0.001); urinary GH was correlated with serum (*p* < 0.05). However, despite being significantly correlated, it is clear from the large differences in absolute concentration in the three media that the appearance of serum GH due to exercise is different from that of the appearance of salivary and urinary GH. This aspect of compartmental exchanges is very difficult to define and to investigate. Differences in any analyte concentration in different compartments are to be expected between different media, and hence the same medium should be used where the same ‘pattern of response’ can be tracked.

**Conclusions:**

The results suggest that urinary and saliva sampling cannot substitute for venous sampling with respect to exercise-induced changes in GH concentration. The use of the analyses in these three areas may be appropriate for further investigation.

## Key Points

1. Traditionally, analysis of the exercise-induced growth hormone (GH) response in athletes has required venous blood sampling.
2. Elite athlete involvement in research is often dependent on less invasive methods of assessment and so the current study has investigated alternatives to venous sampling in athletes.
3. The findings from this study suggest that it may be possible to use blood, saliva and urine interchangeably, in assessing GH secretion in exercise, but confirmation of this will require further investigation.

## Background

For many decades, growth hormone (GH) was believed to have little or no physiological consequence beyond puberty. Today, it is known that growth hormone has a host of functions including growth, repair, substrate regulation [1], maintenance of a healthy body composition, maintenance of exercise capacity and a role in immune function [2]. GH has a considerable effect on the liver, the site of insulin-like growth factor (IGF-1) secretion which is the main hormone linked to GH secretion.

Growth hormone is released in response to physical exercise. The highest transient GH concentrations result from high-intensity exercise (that sustained for 30 s); sustained rises in GH occur where exercise is at an intensity equivalent to lactate threshold or above and is sustained for more than 10 min [3–6]. Exercise also has the ability to override the normal pulsatile secretion of GH [7]. Many of its exercise-induced roles are as yet unknown but recent evidence suggests it may result in post-exercise lipolysis [8]. There has been speculation that growth hormone may potentiate adaptation resulting from exercise and hence this, along with its role in substrate manipulation and in immune function, makes the exercise-induced GH response (EIGR) a target of interest for further research.

The traditional method of venous sampling in individuals, being more invasive, often leads to a reduced number of willing participants. Thus, the viability of less invasive alternatives such as urine and saliva sampling is tested here on athletes. GH concentrations in different media such as saliva and urine in comparison with that in the general circulation after exercise have not been previously investigated.

The aim of this study was to examine changes of GH in saliva, urine and serum after moderate-intensity exercise of moderate duration and to assess the validity of different sample media (saliva and urine) in comparison with analyte measurement in the blood.

## Methods

### Participants

Healthy male subjects (*N* = 11) were recruited from local running clubs and university students and provided signed informed consent. The participants were aged 26 ± 5 years, with those under 18 or over 35 years being excluded, and their characteristics are provided in Table 1. Ethical approval was granted by the Ethics Committee of the School of Sport and Education, Brunel University, and the tenets of the Helsinki Declaration were observed throughout.

**Table 1:** Participant characteristics

	Mean ± SD
Age (years)	26.0 ± 5.0
Height (cm)	179.0 ± 7.0
Body mass (kg)	76.5 ± 9.3
VO_2peak_ (mL kg^−1^ min^−1^)	57.0 ± 6.0
LT (% VO_2peak_)	79.0 ± 6.0

*LT* lactate threshold; *N* = 11

### Protocol

Participants undertook an incremental exercise test on the treadmill to determine lactate threshold and VO_2peak_. After a 5-min warm-up (10 kph), a suitable starting speed was chosen and participants ran at this speed for 4 min then straddled the treadmill for 30 s, while an earlobe capillary blood sample was obtained. The speed of the treadmill was then increased by 0.5 km h^−1^, and participants ran at this speed for 4 min. This process was repeated for a total of five to eight stages, the test protocol taking between 22 and 35.5 min. The criteria for the last stage was a respiratory exchange ratio (RER) consistently at, or above, 1.00. Heart rate (HR) was recorded during the last 30 s of each stage using a Polar heart rate monitor (Polar S610i, Polar Electro Oy, Finland). At the end of the last 4-min stage, a final capillary blood sample was taken as before. Participants then completed 1 min at the same speed and gradient. Thereafter, the gradient of the treadmill was increased by 1 % every minute until (1) volitional exhaustion, (2) a plateau in VO_2_ or (3) an RER greater than 1.15 and HR within five beats min^−1^ of age-predicted maximum [9]. VO_2peak_ was taken as the highest average value for VO_2_ over a 30-s period. Oxygen consumption (VO_2_) and carbon dioxide production (VCO_2_) during exercise were assessed using an online gas analyser (Oxycon Pro, Jaeger, Germany). Prior to every trial, the gas analysers were calibrated using known concentrations of CO_2_ and O_2_ (Air Products Ltd, London) and the flow-volume sensor was calibrated using a 3-litre syringe; giving rise to a CV of <2.0 %.

Within 5–8 days after this test, participants returned to the laboratory to complete a 40-min run at 5 % below the speed at lactate threshold (main trial). In an attempt to standardise the condition of each participant, they were asked to maintain a similar dietary regimen before each trial. In addition, a 3-day food diary was completed and was analysed for percentage of energy derived from carbohydrate, fat and protein using Dietplan6 (Forestfield Software Ltd, UK).

Participants were instructed to fast from 22:00 the night before the test but were allowed to consume water ad libitum. They reported to the laboratory between 07:30 and 08:00; a mid-stream urine sample was collected, height and body mass were recorded and a HR monitor was fitted. Participants rested in a seated position for 10 min before an unstimulated, timed saliva sample and then an 8-mL venous blood sample was collected into a Vacutainer (Becton Dickinson). The sample taken before the exercise was designated ‘Pre-Ex’.

A 5-min warm-up at a self-selected speed (average 10 kph), which was slower than the speed for the main trial, was completed before the 40-min run at 5 % below lactate threshold. Water (200 mL) was consumed during the warm-up period and again during the first 10 min of exercise. As mentioned in the ‘Background’ section, sustained rises in GH occur where exercise is at an intensity equivalent to lactate threshold or above and the performance is sustained for more than 10 min [4–6]. Accordingly, moderate exercise was investigated because it fits with lactate threshold. (That is to say a sustained rise in GH, rather than a transitory peak, requires exercise for at least 10 min above threshold pace). Forty minutes was selected because this corresponds to a good 10-km time and a 10-km pace is generally associated with lactate threshold. The key was a standardised time at just below threshold and, since it was just below threshold, the time was extended to ensure greater chances of achieving steady state which can be more difficult to achieve at, or approaching, threshold pace.

A respiratory gas facemask was worn throughout the trial and respiratory gases were analysed in the last 2 min of every 10 min to monitor exercise intensity; HR was recorded every 10 min. On completion of the main trial, participants returned to a seated position; saliva, venous blood and urine were collected in that order, within <15 min from the start of saliva collection, at (Post-Ex) and after 30 min of rest (Post-Ex 30); saliva and blood were also collected after 60 min of rest (Post-Ex 60). Participants consumed 200 mL of water after the collection of each saliva sample. All samples were centrifuged, aliquotted and stored within 2 h of the tests being completed.

#### Saliva Sampling

Participants were instructed not to have brushed their teeth or drunk anything 30 min prior to each sample. Participants rinsed out their mouths with water and unstimulated, timed (over five min), saliva samples were collected into pre-weighed plastic tubes via drooling, to avoid a contribution from the parotid gland [10, 11], and were immediately frozen at ≤−20 °C. Subsequently, samples were thawed to break down mucopolysaccharides that can interfere with pipetting [12]. The samples were weighed and centrifuged at 1000*g* for 15 min at 20 °C and the clear part of the saliva was aliquotted to multiple Eppendorf tubes and frozen at ≤−20 °C until assay. Freeze-thaw cycles were kept to a minimum. Immediately prior to assay, the saliva was centrifuged again at 1000*g* for 10 min at 20 °C to remove any remaining particulate matter. For the estimation of saliva flow rate (saliva_fr_), it was assumed that saliva density was 1 g mL^−1^ as suggested by Walsh et al. [13]. Saliva_fr_ was calculated by dividing the sample volume (mL) by the time (min) taken to produce it. GH secretion rate (pg min^−1^) was calculated by multiplying the absolute GH concentration (pg mL^−1^) by saliva_fr_ (mL min^−1^) [14].

#### Blood Sampling

Venous blood was collected via venepuncture of an antecubital vein into a vacutainer tube (Becton, Dickinson). Serum (no anti-coagulant) was allowed to clot at room temperature for at least 30 min before being centrifuged at 1000*g* for 12 min at 20 °C. Serum was aliquoted into multiple Eppendorf tubes and frozen at ≤−20 °C until assay. Serum GH is remarkably stable, remaining so for 24 h at room temperature [15, 16]. Earlobe capillary samples were collected into 1.7 mL haemolysing solution and left at room temperature until assayed enzymatically for lactate (Biosen C-lineSport, EZF Diagnostics, Germany).

#### Urine Collection

As described, participants were well hydrated throughout the study to ensure urine production. Urine was collected mid-stream into plastic cups before being transferred into centrifuge tubes, spun at 1000*g* for 12 min at 12 °C to remove particulate, then aliquoted to multiple Eppendorf tubes and stored at ≤−20 °C until assay, which was undertaken within 6 weeks.

#### Sample Analysis

For all assays, samples from the same participant were analysed in the same assay and measured in duplicate.

Serum GH was analysed via ELISA (Diagnostic Systems Laboratories, Texas) in accordance with the manufacturers’ instructions. Optical density was measured using a Titertek MS2 microplate reader. Assay sensitivity was 0.3 ng mL^−1^; intra- and inter-assay CVs were <5 and <7 %, respectively.

Salivary and urinary GH concentration was measured using the same high-sensitivity ELISA kits (Diagnostic Systems Laboratories). For the salivary GH assay, standards and controls were diluted using mock saliva (4.2 g NaOH, 0.5 g NaCl and 0.2 g KOH in 100 mL distilled H_2_O. This mixture was diluted 1:10 in distilled water prior to use. The assays were carried out according to the manufacturer’s instructions. Optical density was determined using a Titertek MS2 microplate reader. Assay sensitivity was 0.66 pg mL^−1^, intra- and inter-assay CV was <10.5 %.

Salivary GH concentrations were corrected for saliva_fr_ as described above. Urinary GH concentrations were corrected for creatinine (Crt) excretion to account for diuresis. Urinary Crt was measured using a colorimetric activity assay (R&D Systems, UK). A standard curve was included with each assay. Urinary GH was calculated as pg mol^−1^ Crt and the intra- and inter-assay CV were ≤3.5 and ≤5.5 %, respectively.

#### Data Analysis

Normality was tested by examining normal plots of residuals in ANOVA models. Residuals were regarded as normally distributed if Kolmogorov-Smirnov tests were not significant [17]. Data were log_10_ transformed to better approach normality. One-way repeated measures ANOVA was used to indicate significant changes over time; when the assumption of sphericity was violated, the *p* value was adjusted using the Greenhouse-Geisser correction [17]. Where appropriate, paired-sample *t* tests using the Bonferroni adjustment were used to indicate changes compared to Pre-Ex. Associations between the concentration of GH in saliva and urine with those in serum were assessed by calculating Pearson’s (*r*) or Spearman’s rho (rs) correlation coefficient depending on whether the variables were normally distributed or not. Data were analysed using SPSS for windows, version 13.0 (SPSS Inc., Chicago, USA).

## Results

During the 40-min exercise bout, mean ± SD HR was 163 ± 9 beats min^−1^, VO_2_ was 44 ± 5 mL kg^−1^ min^−1^ and % VO_2peak_ was 77 ± 5 %. The average daily energy derived from carbohydrate, fat and protein (mean ± SD) was 57 ± 9 %, 29 ± 8 % and 14 ± 2 %, respectively. A complete data set for urine samples could not be obtained at Post-Ex 60, and thus, these measurements were confined to Post-Ex 30. Crt was not detected in one Pre sample.

Changes in serum, salivary and urinary GH in response to the exercise are presented in Table 2. Serum GH changed significantly over time (*F* = 26.27, *p* < 0.001); at Post-Ex, serum GH was increased by 4.5-fold. Absolute salivary concentration of GH (pg mL^−1^) changed significantly over time (*F* = 33.99, *p* < 0.001); it was increased by 33-fold at Post-Ex, 40.5-fold at Post-Ex 30 and 35-fold at Post-Ex 60 compared with Pre-Ex. Similar results were observed when salivary concentrations were corrected for saliva_fr_ (pg min^−1^). Increases of 58.5-fold, 72-fold and 50-fold were observed at Post-Ex Post-Ex 30 and Post-Ex 60, respectively, compared with Pre-Ex. The concentration of urinary GH, both absolute and corrected for Crt excretion, changed significantly over time (*F* = 12.27, *p* < 0.001 and *F* = 7.12, *p* < 0.01, respectively). The absolute concentration of urinary GH was increased by 15.5-fold at Post-Ex and 25-fold at Post-Ex 30 compared with Pre-Ex. The concentration of urinary GH relative to Crt excretion was increased by 23-fold at Post-Ex and 52-fold at Post-Ex 30 compared with Pre-Ex. Despite rather weak correlations, serum GH was significantly correlated with salivary GH before and after exercise for both the absolute concentration and corrected for saliva_fr_ (rs = 0.412, *p* < 0.01 and rs = 0.392, *p* < 0.01, respectively). Serum GH was significantly correlated with the absolute concentration of urinary GH and urinary GH corrected for Crt excretion (rs = 0.434, *p* < 0.05 and *rs* = 0.484, *p* < 0.01, respectively).

**Table 2:** Serum, salivary and urinary GH in response to moderate-intensity exercise

	Pre-Ex	Post-Ex	Post-Ex 30	Post-Ex 60
Serum (ng mL^−1^)	1.8 ± 1.2	8.2 ± 1.0***	2.1 ± 0.5	0.7 ± 0.2
Saliva (pg mL^−1^)	0.6 ± 0.5	19.7 ± 6.0***	24.3 ± 5.8***	20.9 ± 8.3***
Saliva_fr_ (pg min^−1^)	0.2 ± 0.2	11.7 ± 3.8***	14.4 ± 4.8***	10.0 ± 4.2***
Urine (pg mL^−1^)	5.1 ± 1.9	79.2 ± 32.3**	128.0 ± 83.0*	
Urine (pg mol^−1^ Crt)	0.4 ± 0.2	9.3 ± 4.8**	20.9 ± 16.3*	

Data are mean ± SEM (*N* = 11), significantly different from Pre-Ex

*Saliva*_*fr*_ saliva flow rate, *Crt* creatinine

*p < 0.05; **p < 0.01; ***p < 0.001

## Discussion

The present study is the first to investigate the effect of an acute bout of exercise on the concentration of GH simultaneously in serum, urine and saliva. Dietary analysis (individual data not shown) revealed that participants derived a similar percentage of average daily energy intake from carbohydrate, fat and protein. Thus, the contribution of differences in dietary composition can be considered to be minimal.

A significant correlation between serum and salivary GH before and after the bout of exercise was observed in the present study on males. Rantonen et al. also reported a relationship between serum and salivary GH at rest, but only in women [18]. The report of a significant correlation between serum and urinary GH before and after exercise is a novel finding.

Despite being significantly correlated, it is clear from the results (Table 2) that the serum GH response to exercise is different from that of the salivary and urinary GH responses. First, serum GH is in the nanogram range, whereas urine and saliva GH is in the picogram range. A lower concentration of GH in saliva compared to serum has been observed [19]. It is also known that, in the absence of disease, approximately 0.01 % of secreted GH is excreted into the urine, which would explain the low urinary concentrations [20].

Secondly, serum GH peaked at the end of the exercise and returned to baseline by 30 min post-exercise. Salivary GH increased immediately after exercise, peaked at 30 min post-exercise and remained above baseline until 1 h post-exercise. Urinary GH was also increased immediately after exercise and was increased further at 30 min post-exercise. Hence, the pattern of change in systemic GH was not reflected in saliva or urine in the present study. The metabolic clearance rate of GH might be a factor. In healthy humans, the clearance of recombinant human (rh) GH appears to be inversely related to plasma GH concentration [21]. Therefore, it is possible that, in the present study, when serum GH was high immediately post-exercise, clearance via processes in the kidney and saliva may have been low. As serum GH decreased in the hour after exercise, metabolic clearance rate may have increased leading to the increased appearance of GH in saliva and urine. Another possible explanation concerning urinary excretion of GH is the linear relationship between metabolic clearance rate and glomerular filtration rate observed by Haffner et al. [21]. It is tempting to speculate that, if the metabolic clearance rate of GH increased in the hour after exercise, the filtration rate of GH would lead to an increase in the appearance of GH in urine.

Findings from this study suggest that salivary and urinary GH concentrations are not viable alternatives to serum measures of GH concentration as an index of systemic GH concentration. It would have been interesting to compare different intensities/duration of exercise, to see if any correlations (comparison of different sample media compared to blood measurement) occurred.

## Conclusions

This is the first study to measure simultaneous changes in salivary, urinary and systemic concentration of GH, before and after an acute bout of exercise. Serum, salivary and urinary GH were significantly increased immediately after exercise, but did not have a similar pattern of change over time. GH concentrations were markedly higher in serum than in saliva and urine. The appearance of GH in saliva and urine after exercise may be related to its clearance from the circulation. There appears to be a paucity of research examining the mechanisms behind the entry of GH into saliva and urine after exercise.

Further research, perhaps using radio labelling with I^125^, would greatly enhance our understanding of GH clearance during exercise, but understanding clearance after exercise may prove more problematic.

## Abbreviations

Crt, creatinine; EIGR, exercise-induced growth hormone response; GH, growth hormone; HR, heart rate; RER, respiratory exchange ratio; VCO_2_, carbon dioxide production; VO_2_, heart oxygen consumption
